# Baculovirus Expression and Functional Analysis of Vpa2 Proteins from *Bacillus thuringiensis*

**DOI:** 10.3390/toxins12090543

**Published:** 2020-08-22

**Authors:** Oihane Simón, Leopoldo Palma, Ana Beatriz Fernández, Trevor Williams, Primitivo Caballero

**Affiliations:** 1 Institute for Multidisciplinary Research in Applied Biology, Universidad Pública de Navarra, 31006 Pamplona, Navarra, Spain; anabeatriz.fernandez@unavarra.es (A.B.F.); pcm@unavarra.es (P.C.); 2 Instituto Académico Pedagógico de Ciencias Básicas y Aplicadas, Centro de Investigaciones y Transferencia de Villa María (CITVM-CONICET), Universidad Nacional De Villa María, Villa María, Córdoba 5900, Argentina; palma.leopoldo@gmail.com; 3 Departamento de Investigación y Desarrollo, Bioinsectis SL, Polígono Industrial Mocholi Plaza Cein 5, Nave A14, 31110 Noain, Navarra, Spain; 4 Instituto de Ecología AC, Xalapa, Veracruz 91073, Mexico; trevor.inecol@gmail.com

**Keywords:** vegetative insecticidal proteins, Vpb/Vpa, entomopathogen, ADP-ribosyltransferase, recombinant baculovirus, lepidopteran cells, biopesticides, broad spectrum, Vip1/Vip2

## Abstract

The mode of action underlying the insecticidal activity of the *Bacillus thuringiensis* (Bt) binary pesticidal protein Vpb/Vpa (formerly Vip1/Vip2) is uncertain. In this study, three recombinant baculoviruses were constructed using Bac-to-Bac technology to express Vpa2Ac1 and two novel Vpa2-like genes, Vpa2-like1 and Vpa2-like2, under the baculovirus p10 promoter in transfected Sf9 cells. Pairwise amino acid analyses revealed a higher percentage of identity and a lower number of gaps between Vpa2Ac1 and Vpa2-like2 than to Vpa2-like1. Moreover, Vpa2-like1 lacked the conserved Ser-Thr-Ser motif, involved in NAD binding, and the (F/Y)xx(Q/E)xE consensus sequence, characteristic of the ARTT toxin family involved in actin polymerization. Vpa2Ac1, Vpa2-like1 and Vpa2-like2 transcripts and proteins were detected in Sf9 culture cells, but the signals of Vpa2Ac1 and Vpa2-like2 were weak and decreased over time. Sf9 cells infected by a recombinant bacmid expressing Vpa2-like1 showed typical circular morphology and produced viral occlusion bodies (OBs) at the same level as the control virus. However, expression of Vpa2Ac1 and Vpa2-like2 induced cell polarization, similar to that produced by the microfilament-destabilizing agent cytochalasin D and OBs were not produced. The presence of filament disrupting agents, such as nicotinamide and nocodazole, during transfection prevented cell polarization and OB production was observed. We conclude that Vpa2Ac1 and Vpa2-like2 proteins likely possess ADP-ribosyltransferase activity that modulated actin polarization, whereas Vpa2-like1 is not a typical Vpa2 protein. Vpa2-like2 has now been designated Vpa2Ca1 (accession number AAO86513) by the *Bacillus thuringiensis* delta-endotoxin nomenclature committee.

## 1. Introduction

Bacteria have developed numerous pesticidal proteins and effectors to target the actin cytoskeleton [1]. The cytoskeleton is a structure that helps maintain the shape and internal organization of cells and provides mechanical support that enables cells to carry out essential functions. It consists of three major classes of elements that interact with each other and differ in size and protein composition [2,3]. Microtubules, composed of tubulin, form the largest type of filaments, followed by intermediate filaments that vary in composition, while actin filaments are the smallest type [2]. Interactions between microtubules and actin underlie many fundamental processes for which dynamic cellular asymmetries need to be established and maintained [3]. Cytoplasmic microtubules are essential for the establishment of cell polarity and direct the transport of intracellular vesicles over long distances [1]. Both F-actin filaments and microtubules are highly dynamic structures, which are regulated by a large number of binding proteins, often the effectors of intracellular and extracellular signaling pathways. Bacterial toxins and effectors target the actin cytoskeleton by interfering with the endogenous regulation of the cytoskeleton or by directly modifying the actin molecule [4,5]. Such cytoskeletal regulation can be achieved through ADP-ribosylation, glycosylation, proteolysis, adenylation, deamidation and transglutamination [1]. Various bacterial toxins induce actin ADP-ribosylation. Members of the ADP-ribosyltransferase toxins family confer a single post-translational modification, the addition of ADP-ribose, on various eukaryotic substrates, including RHO proteins (for *Clostridium botulinum* C3 exoenzymes), heterotrimeric G proteins (for *Vibrio cholerae* cholera toxin and *Bordetella pertussis* pertussis toxin) and actin (for *C. botulinum* C2 toxin). These modifications often inhibit normal eukaryotic protein function [6]. The prototype of these toxins is the family of binary actin-ADP-ribosylating toxins, comprising of a receptor ligand and an enzymatically active toxic component, which performs ADP-ribosylation of the Arg177 residue of G-actin [7,8,9,10], resulting in the depolymerization of F-actin filaments and destruction of the actin cytoskeleton [1,11]. Toxin-induced depolymerization of actin causes dramatic effects on the physiology and function of target cells.

Cell protrusions are formed by microtubule structures that form a dense network at the surface of epithelial monolayers and play an important role in the infectivity and propagation of bacterial pathogens [12]. As mentioned above, actin microfilaments and microtubule structures regulate each other in a dynamic fashion. Thus, ADP-ribosylation of actin, which results in depolymerization of F-actin, affects the regulation of the dynamic behavior of microtubules [13] and causes the formation of tubulin protrusions [12]. This microtubule-based network of protrusions on the surface of epithelial cells has major consequences for the adherence of bacteria, as toxin-producing bacteria adhere strongly to epithelial cells [12]. Additionally, increasing activity of the actin-ADP-ribosylating toxin results in increased dissemination of bacteria [12,14].

*Bacillus thuringiensis* is an entomopathogenic bacterium that produces a diversity of insecticidal proteins, including vegetative pesticidal proteins that are secreted into the culture medium, rather than sequestered into the parasporal crystal like the many Cry and Cyt proteins produced by this bacterium during sporulation [15,16,17]. Vegetative pesticidal proteins are divided into three families (Vip, Vpa and Vpb) according to their pairwise percentage of amino acid identity and insecticidal specificities [18,19]. Vip, formerly Vip3, are multi-domain proteins that have no sequence similarity to Vpb or Vpa and exhibit toxic activity against a wide variety of Lepidoptera, with a mode of action, which seems to resemble that of Cry proteins [15]. Vpb are proteins related to the binding component of binary toxins, such as Vip1 and Vip4. Finally, Vpa proteins are believed to be related to the ADP-ribosyltransferase active component of binary toxins such as Vip2 (from the Vip1/Vip2 toxin) [18,19], which have been reported to be toxic to some species of Coleoptera and Hemiptera [15,16].

The Vpb1/Vpa2 binary toxin (Vip1/Vip2) seems to be an example of a classical A+B type binary toxin, such as the potent binary toxins produced by *Vibrio cholerae*, *Clostridium* spp. or *Bacillus anthracis* [20]. Vpb1 shares a moderate sequence identity with the binding component C2-II of the C2 *C. botulinum* toxin (29%) and the Ib component of iota-toxin from *C. perfringens* (31%) [15]. In contrast, Vpa2 exhibits both sequence and structural similarity (>30%) with the enzymatic domain of toxin CdtA from *C. difficile* and the iota-toxin domain Ia from *C. perfringens*, both of which possess ADP-ribosyltransferase activity that targets actin, leading to cytoskeletal disorders and cell death [16,20,21,22]. Moreover, the Vpa2 crystal structure and its NAD+ complex form are compatible with this mechanism of action [16,20], although the actual mode of action remains unproved. The proposed mechanism of action of these toxins involves the proteolytic activation of the cell-binding B precursor (Vpb1) and its monomeric interaction with the cell surface receptor(s), followed by formation of homoheptamers that subsequently translocate the toxic A component (Vpa2) into the cytoplasm through acid endosomes [20]. Once inside the cytoplasm, the A component destroys filamentous actin, likely by mono-ADP-ribosylation of the Arg177 residue of G-actin, blocking its polymerization and leading to cell death by cytoskeletal disarrangement [1,15,16,23,24]. In line with this idea, it has been suggested that Vpb1 may facilitate the binding of Vpa2 toxin to intestinal brush border membranes in coleopteran larvae [25].

The molecular mechanism underlying Vpb1/Vpa2 insecticidal activity is not well understood, and the role of Vpa2 in cytoskeletal disruption has not been examined. Vpb1 acts as a binding receptor for Vpa2 [25]. However, few studies have focused on the biological characterization of Vpa2 proteins, as they are difficult to clone and produce due to their cytotoxicity. Vpb1/Vpa2 shows high insecticidal activity against some coleopteran pests [15,26] and the sap-sucking aphid pest, *Aphis gossypii* (Hemiptera) [15,27]. This suggests that Vpb1 might bind to specific cell receptors present in those insects. In the present study, we aimed to demonstrate that Vpa2 proteins from *B. thuringiensis* display ADP-ribosyltransferase activity. For this, three recombinant baculoviruses were constructed using Bac-to-Bac technology to express Vpa2 proteins. Filamentous actin (F-actin) is required for progeny production in alphabaculoviruses (nucleopolyhedroviruses of Lepidoptera) [28], as it is essential for nucleocapsid morphogenesis and assembly within the nucleus [28,29,30]. F-actin is a genus-wide requirement for the replication of these viruses in lepidopteran hosts [28,30]. Therefore, if Vpa2 proteins block actin polymerization the recombinant baculoviruses expressing these proteins are likely to reduce occlusion body (OB) progeny production. In a previous study, *B. thuringiensis* isolates were screened for the presence of Vpa2 genes [31]. Two possible novel Vpa2 proteins were identified: Vpa2-like1 and Vpa2-like2. Vpa2Ac1 was used as a positive control (a reference treatment), which was believed to exhibit ADP-ribosyltransferase activity [15,16,25]. The effects of these proteins on lepidopteran cells and larvae, and on virus production were evaluated.

## 2. Results

### 2.1. Vpa2 Protein Sequence Analysis

**Figure 1:**
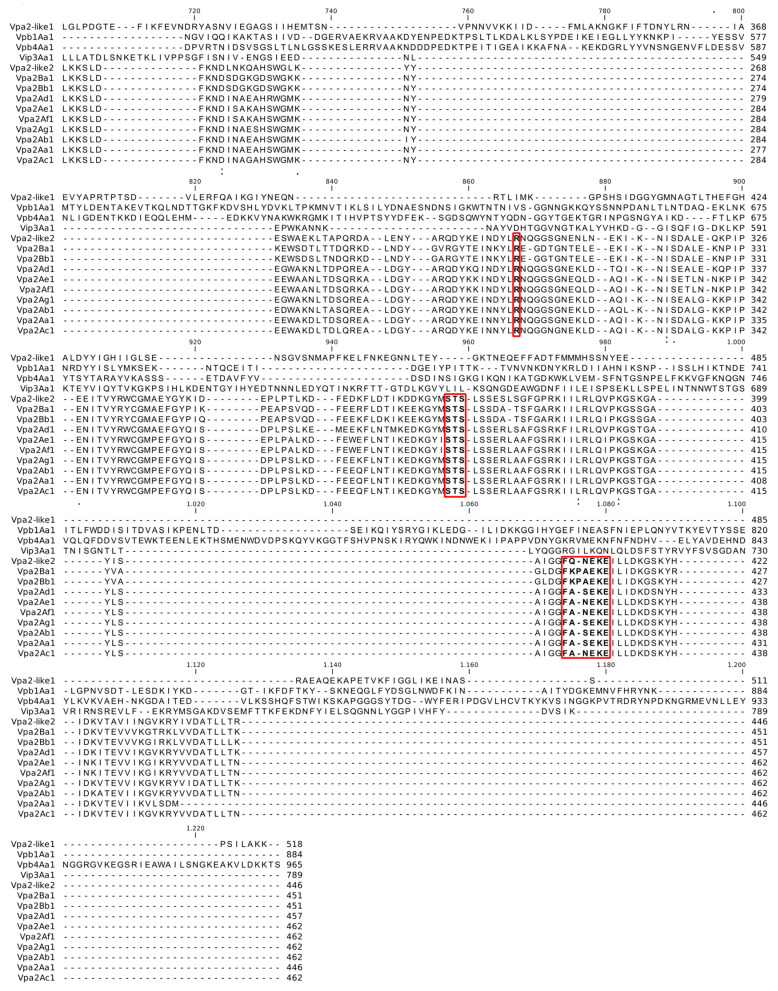
Global alignment of Vpa2Ac1, Vpa2-like1 and Vpa2-like2 proteins. The Ser-Thr-Ser motif is marked with a red rectangle (STS). The (F/Y)xx(Q/E)xE consensus sequence, characteristic of the ARTT toxin family, is marked within a red rectangle and in bold. Finally, the conformational flexibility of the ligand binding pocket (R) is within a red rectangle and shown in bold.

The Vpa2Ac1, Vpa2-like1 and Vpa2-like2 genes from the TF037.2, H001.5 and H026.2 isolates of *B. thuringiensis* [32] had open reading frames of 1389, 1557 and 1341 bp, encoding proteins of 462, 518 and 446 amino acids with a molecular mass of 52.4, 58.6 and 51.1 kDa, respectively. Pairwise comparisons revealed that Vpa2Ac1 and Vpa2-like2 exhibited a higher percentage of pairwise identity between one another than to Vpa2-like1. Vpa2Ac1 and Vpa2-like2 presented 63% pairwise identity, 292 identical and 171 different amino acids. However, Vpa2-like1 had just 16% of the pairwise identity with 89 identical and 456 different amino acids compared with Vpa2Ac1, and 16.6% of the identity, 90 identical and 451 different amino acids when compared with Vpa2-like2. Vpa2Ac1 and Vpa2-like2 were more closely related to one another than to Vpa2-like1. Those differences dramatically altered the predicted conserved domain sequences present in each protein (Figure 1).

Vpa family proteins and Vpa2-like2 both presented a conserved Ser-Thr-Ser (STS) motif (indicated by a rectangle in Figure 1), an active site that plays an important role in both catalysis and structure, which is involved in NAD binding. The STS motif was absent in Vpa2-like1. Similarly, the (F/Y)xx(Q/E)xE consensus sequences (marked with a rectangle and in bold residues in Figure 1), characteristic of the ARTT toxin family, was absent in Vpa2-like1. This motif acts in the depolymerization of the actin cytoskeleton. Finally, the conformational flexibility of the ligand binding pocket (R, marked with a rectangle in Figure 1) was also absent in Vpa2-like1, which is involved in the closure of the active site pocket upon ligand binding. The Vpa2 superfamily motif was present in Vpa2Ac1, Vpa2-like1 and Vpa2-like2, but two Vpa2 motifs were present in Vpa2-like2 and Vpa2Ac1, unlike Vpa2-like1 that has only one such motif. The Vpa2 superfamily motif was located between residues 86–239 (E-value of 4.32 × 10^−10^), 70–277 (E-value of 6.38 × 10^−11^) and 57–246 (E-value of 1.10 × 10^−13^) in Vpa2Ac1, Vpa2-like1 and Vpa2-like2, respectively, whereas the Vpa2 motif, present only in Vpa2Ac1 and Vpa2-like2, was located between residues 264-460 (E-value of 1.66 × 10^−76^) and 248–444 (E-value of 9.04 × 10^−62^). In contrast, in Vpa2-like1 an ATLF superfamily motif was present between residues 282–507 (E-value of 4.60 × 10^−11^), which is the anthrax lethal factor N-terminal domain. However, the C-terminal domain is the catalytically active domain, whereas the N-terminal domain is likely to be inactive.

**Figure 2:**
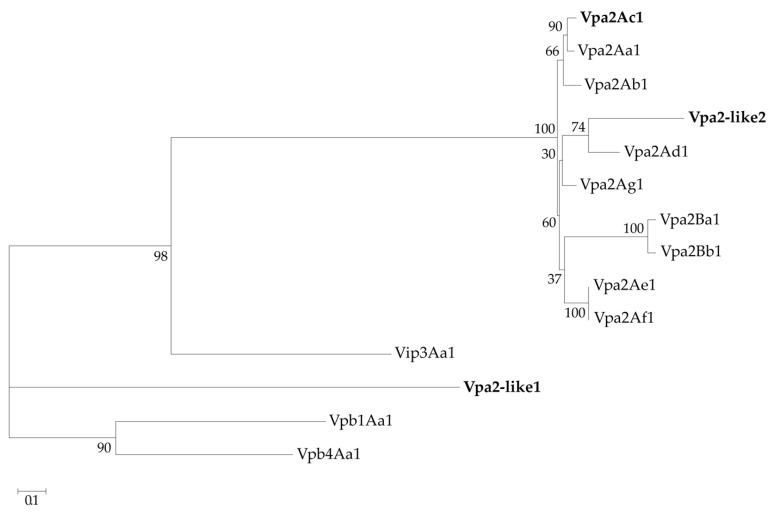
Phylogenetic analysis of *Bacillus thuringiensis* pesticidal proteins. Vpa2Ac1, Vpa2-like1 and Vpa2-like2 proteins (in bold) were compared with other well-characterized Vip, Vpa and Vpb proteins. The maximum likelihood tree was constructed based on a LG amino acid substitution model. Bootstrap values (10,0000 replicates) are indicated next to the nodes.

In addition, the amino acid sequences of Vpa2-like2 and Vpa2-like1 were compared with well-known Vpa2 proteins, including Vip3Aa1 and Vpb1Aa1 and Vpb4Aa1 proteins as outgroups available at https://www.bpprc.org/, and were analyzed phylogenetically by maximum likelihood estimation (Figure 2). Unlike Vpa2-like1, Vpa2-like2 was located within the Vpa2 cluster. Compared with other Vpa2 proteins, Vpa2-like2 had pairwise amino acid identity, which ranged between 56% and 68%, whereas the corresponding identities with the other pesticidal proteins (Vip3, Vpb1 and Vpb4) were less than 9%. In contrast, Vpa2-like1 differed markedly from the Vpa2 class proteins described to date, and also differed from the Vip3, Vpb1 and Vpb4 classes. This suggested that the Vpa2-like1 protein might be a Vpa2 class member and could be a new class of insecticidal protein that merited characterization of its insecticidal activity (Figure 2), Therefore, Vpa2-like2 was considerably closer to the Vpa2 cluster than Vpa2-like1.

### 2.2. Transfection of Sf9 Cells with Recombinant Bac-polh-Vpa2Ac1, Bac-polh-Vpa2like1, Bac-polh-Vpa2like2 and Bac-polh-Ø virus DNAs

**Figure 3:**
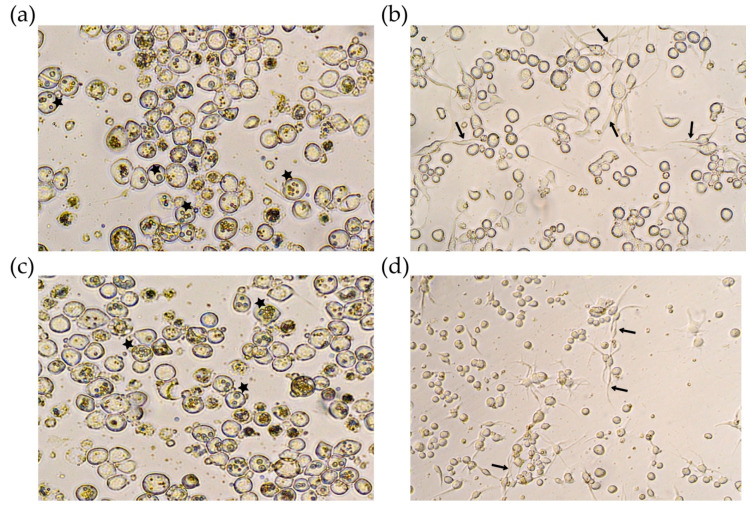
Optical microscope images at 400× of Sf9 cells infected with (**a**) recombinant bacmid without any gene of interest (Bac-polh-ø) used as a positive control, and recombinant bacmids expressing (**b**) Vpa2Ac1 (Bac-polh-Vpa2Ac1), (**c**) Vpa2-like1 (Bac-polh-Vpa2like1) and (**d**) Vpa2-like2 (Bac-polh-Vpa2like2). Black arrows indicate cell protrusions and stars indicate cells containing OBs. In figure (**a**) and (**c**) most of cells presented normal form with OBs within cells (stars), while in figure (**b**) and (**d**) cells showed deformations and appeared to have lysed or become elongated, with numerous protrusions (arrows).

Transfected cells were checked daily, and five days after infection, most of the cells infected with Bac-polh-Ø and Bac-polh-Vpa2like1 showed OBs present in the cell nuclei (Figure 3). However, cells transfected with Bac-polh-Vpa2Ac1 and Bac-polh-Vpa2like2 showed deformations and some cells appeared to have lysed or become elongated. Many cells had protrusions and began to lengthen and took on an irregular atypical appearance. No OBs were observed in those transfected cells (Figure 3).

Infected cells were recovered and lysed to obtain viral OBs. DNA extraction was performed on these OBs. Restriction endonuclease (REN) analyses showed that viral DNA profiles from Bac-polh-Ø and Bac-polh-Vpa2like1 transfections were the same as those of the original inocula (Figure S1b and Figure S2a). In contrast, Bac-polh-Vpa2Ac1 and Bac-polh-Vpa2like2 DNA appeared to be degraded (Figure S2a). The supernatant containing budded virions (BVs) was used for injection in *Spodoptera exigua* fourth instar larvae to produce large quantities of OBs. Injection of Bac-polh-Ø and Bac-polh-Vpa2like1 BVs resulted in a similar prevalence of larvae mortality (range between 86 and 95%), whereas no virus-killed larvae were obtained following injection with Bac-polh-Vpa2Ac1 or Bac-polh-Vpa2like2 BVs. REN treatment of Bac-polh-Ø and Bac-polh-Vpa2like1 OBs resulted in the characteristic profiles of each virus (Figure S2b), which corresponded to the original inoculated viruses (Figure S1b). PCR and sequence analysis also showed sequences that were identical to those of the original inocula (Figure S1b).

### 2.3. Detection of Vpa2 Transcripts and Proteins in Transfected Cells

Vpa2 transcripts were detected 24 h post infection (hpi) in cells infected with recombinant Bac-polh-Vpa2Ac1, Bac-polh-Vpa2like1 and Bac-polh-Vpa2like2, but not in cells infected with Bac-polh-Ø (Figure S3a). However, the signal of Vpa2Ac1 and Vpa2-like2 transcripts decreased over time. In the Western blot, Vpa2 proteins were successfully detected (Figure S3b), whereas the signal for Bac-polh-Ø was not detectable. However, Vpa2Ac1 or Vpa2-like2 signals were very weak and decreased over time. These findings confirm that the recombinant viruses expressed each of the Vpa2 genes.

### 2.4. Effect of Actin-Modulating Compounds on Viral Transfection

**Figure 4:**
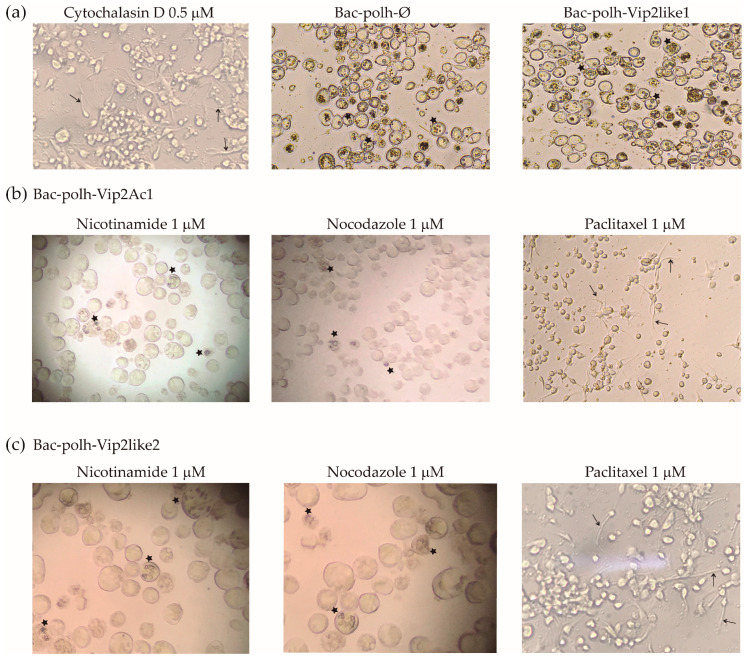
Images of Sf9 cells treated with (**a**) control treatments; cytochalasin D, for cell depolarization control, and Bac-polh-ø and Bac-polh-Vpa2like1 recombinant viruses, as the control for the production of progeny occlusion bodies (OBs), and (**b**) with Bac-polh-Vpa2Ac1 and (**c**) Bac-polh-Vpa2like2 viruses treated with nicotinamide, nocodazole and paclitaxel at 1 µM. Black arrows indicate cell protrusions. Stars indicate cells containing OBs. Cells treated with cytochalasin D showed cell protrusions as those infected with Bac-polh-Vpa2Ac1 and Bac-polh-Vpa2like2 and treated with paclitaxel. Cells infected with Bac-polh-ø and Bac-polh-Vpa2like1 showed high OB production, with characteristic OBs within cells. In contrast, cells infected with recombinant viruses expressing Vpa2Ac1 and Vpa-like2 showed cell protrusions (Figure 3) but when treated with nicotinamide and nocodazole cells recovered their original form and some showed OB production.

Cells treated with nicotinamide, nocodazole or paclitaxel alone, showed normal morphology. Cells infected with recombinant viruses expressing Vpa2Ac1 and Vpa2-like2 proteins showed similar morphology to those incubated with cytochalasin D in the absence of virus infection (Figure 4), in which actin polymerization was inhibited. In contrast, when the transfected cells were incubated with nocodazole and nicotinamide, at all concentrations (Figure 4 shows only the results at 1 µM), cells recovered their original form and OBs were observed in low quantities compared with those produced by Bac-polh-Ø and Bac-polh-Vpa2like1. Infections with Bac-polh-Vpa2Ac1 or Bac-polh-Vpa2like2 did not spread to surrounding cells and only a small number of cells produced OBs (Figure 4). In contrast, in cells infected with Bac-polh-Ø and Bac-polh-Vpa2like1, the number of OBs was similar between cells incubated with or without nocodazole and nicotinamide. When cells were incubated with paclitaxel, transfection with Bac-polh-Ø or Bac-polh-Vpa2like1 resulted in normal morphology and typical production of OBs (data not shown), whereas cells transfected with Bac-polh-Vpa2Ac1 and Bac-polh-Vpa2like2 showed surface protrusions (indicated with arrows in Figure 4) and an absence of OB production (Figure 4).

**Figure 5:**
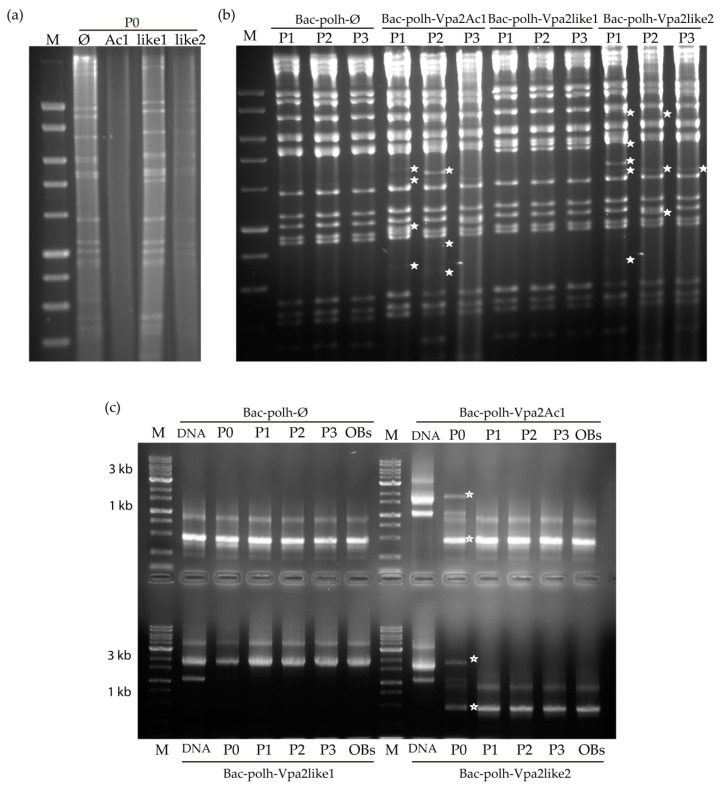
Restriction endonuclease profiles with *Pst*I of (**a**) cell pellets (P0) after transfection of Sf9 cells with recombinant bacmids Bac-polh-ø, Bac-polh-Vpa2Ac1, Bac-polh-Vpa2like1 and Bac-polh-Vpa2like2, treated with nicotinamide in the case of Bac-polh-Vpa2Ac1 and Bac-polh-Vpa2like2 (similar results were obtained with nocodazole) and (**b**) *Pst*I profiles of cell pellets after three passages (P1, P2 and P3) in Sf9 cells of recombinant bacmids Bac-polh-ø, Bac-polh-Vpa2Ac1, Bac-polh-Vpa2like1 and Bac-polh-Vpa2like2 treated with nicotinamide. White stars indicate the new fragments that appeared. The molecular size marker (M) was smart ladder (Stratagene, San Diego, CA, USA). (**c**) PCR analysis of original bacmid DNAs of Bac-polh-ø, Bac-polh-Vpa2Ac1, Bac-polh-Vpa2like1 and Bac-polh-Vpa2like and DNA from cell pellet (P0) after transfection with bacmid DNAs and nicotinamide in the case of Bac-polh-Vpa2like1 and Bac-polh-Vpa2like, and the cell pellets after three passages (P1, P2 and P3) in Sf9 cells of recombinant bacmids Bac-polh-ø, Bac-polh-Vpa2Ac1, Bac-polh-Vpa2like1 and Bac-polh-Vpa2like2 treated with nicotinamide. In the analysis, the OBs obtained after injection of *S. exigua* larvae with budded virions (BVs) at P0, and used in bioassays, were also included. The white stars indicate the two fragments obtained in cell pellets at P0. The molecular size marker (M) was smart ladder (Stratagene).

REN analysis of the inoculum OBs obtained in cell culture (passage one, P0) revealed that Bac-polh-Ø and Bac-polh-Vpa2like1 presented their characteristic profiles, whereas Bac-polh-Vpa2Ac1 and Bac-polh-Vpa2like2 profiles appeared mostly degraded, although some bacmid bands were distinguishable (Figure 5a), in comparison with the profiles obtained in the absence of actin-modulating compounds (Figure S2a). The REN profile was similar to Bac-polh-Ø, and the characteristic band of Bac-polh-Vpa2Ac1 and Bac-polh-Vpa2like2 profiles (asterisks in Figure S1b) seemed to be absent. After three passages in cell culture, the Bac-polh-Ø and Bac-polh-Vpa2like1 REN profiles remained stable and were identical to those of the original inocula. However, the Bac-polh-Vpa2Ac1 and Bac-polh-Vpa2like2 profiles presented clear variations compared to the original profiles, even after a single passage. Specifically, certain restriction fragments were not detectable and new bands appeared (Figure 5b). Moreover, the diagnostic restriction fragment of Bac-polh-Vpa2Ac1 and Bac-polh-Vpa2like2 viruses in the REN profiles (Figure S1) was not visible in the REN profiles of the BVs obtained in the cell culture and OBs produced in larvae.

PCR using primers p10x.F and p10x.R was performed with the DNAs of the original bacmids and the DNAs extracted from BVs and OBs at the different passages (P0-P3; Figure 5c). The original bacmid DNAs all showed the expected sizes, being 500 bp for Bac-polh-Ø, and 1.7 kb for Bac-polh-Vpa2Ac1 and Bac-polh-Vpa2like2 recombinant viruses, and 1.8 kb for Bac-polh-Vpa2like1. This was consistent with the presence of the corresponding genes in the genomes of Bac-polh-Vpa2Ac1, Bac-polh-Vpa2like1 and Bac-polh-Vpa2like2. In the case of Bac-polh-Vpa2like1, the same band was observed in DNAs from BVs and OBs, suggesting that the gene was conserved through successive passages in cells and larvae. However, in DNAs extracted from cell pellets (P0) of Bac-polh-Vpa2Ac1 and Bac-polh-Vpa2like2, the PCR fragment was observed but much less intensely than in the original bacmid DNAs, and the 500 bp band was present that corresponded to the Bac-polh-Ø control bacmid, or to genotypes that had lost the *vpa2Ac1* and *vpa2-like2* genes. This result was in agreement with the weak protein signal present in the SDS-PAGE analysis (Figure S2). In contrast, in the DNAs extracted from cell pellets at different passages and from OBs produced in larvae of Bac-polh-Vpa2Ac1 and Bac-polh-Vpa2like2, the 1.7 kb band was absent and only the 500 bp band was visible. This result suggests that in cell pellets and BVs at P0 some genomes conserved their recombinant gene, but after passage through cells and larvae these bacmids had completely lost their recombinant gene. Similarly, OBs collected from virus-killed larvae that had been injected with Bac-polh-Vpa2Ac1 and Bac-polh-Vpa2like2 BVs showed variations in the REN profiles in comparison with the original inocula and PCR was unable to amplify the recombinant gene sequences (Figure 5c). It appears that Bac-polh-Vpa2Ac1 and Bac-polh-Vpa2like2 were not stable and underwent recombinations, mutations or insertions that modified their REN profiles.

### 2.5. Biological Activity of Recombinant Viruses Expressing Vpa2Ac1, Vpa2-like1 and Vpa-like2

Biological assays were performed on the OBs obtained following injection of *S. exigua* larvae with BVs obtained from the transfection of Sf9 cells with recombinant bacmids Bac-polh-ø, Bac-polh-Vpa2Ac1, Bac-polh-Vpa2like1 and Bac-polh-Vpa2like2, and OBs obtained following the injection of *S. exigua* larvae with BVs from nicotinamide-treated cells in the case of Bac-polh-Vpa2Ac1 and Bac-polh-Vpa2like2 (although PCR analysis showed that Vpa2Ac1 and Vap2-like2 genes were absent in the genomes of the latter two bacmids).

The recombinant bacmid that expressed Vpa2-like1 genes was approximately 4–6-fold less infective than the control virus, in all species tested (Table 1). The LC_50_ values, lethal concentration that kills 50% of the population assayed, for Bac-polh-Vpa2like1 virus were consistently higher than Bac-polh-ø at 2.36 × 10^7^, 1.00 × 10^5^, 3.17 × 10^7^ and 4.83 × 10^6^ OBs/mL, for *M. brassicae*, *S. exigua*, *S. littoralis* and *S. frugiperda*, respectively, indicating that the expression of the Vpa2-like1 protein reduced the insecticidal characteristics of viral OBs.

In contrast, Bac-polh-Vpa2Ac1 and Bac-polh-Vpa2like2 OBs showed differences in pathogenicity among the different species. Bac-polh-Vpa2Ac1 OBs were 1.82-fold and 2.08-fold less pathogenic in *M. brassicae* and *S. exigua* than the Bac-polh-Ø control virus OBs, respectively (Table 1). In contrast, in *S. littoralis* and *S. frugiperda* Bac-polh-Vpa2Ac1 presented similar pathogenicity to the control virus, with LC_50_ values of 1.47 × 10^7^ and 1.03 × 10^6^ OBs/mL, in comparison with those of Bac-polh-Ø of 1.24 × 10^7^ and 8.43 × 10^5^ OBs/mL, respectively. Similarly, Bac-polh-Vpa2like2 activity in *M. brassicae* and *S. littoralis* was similar to the control virus, whereas in *S. exigua* and *S. frugiperda* Bac-polh-Vpa2like2 OBs were 2.08-fold and 1.82-fold less pathogenic than the control virus OBs (Table 1). The genetic variation present in Bac-polh-Vpa2Ac1 and Bac-polh-Vpa2like2 OBs, likely due to mutations and recombination events, affected the pathogenicity of these OBs differently in the different host species tested.

## 3. Discussion

The ADP-ribosyltransferase activity of Vpa2 toxins from *B. thuringinesis* was investigated by constructing recombinant baculoviruses expressing these proteins that were then used to infect lepidopteran cells and larvae. Cells infected by the recombinant bacmid expressing Vpa2-like1 protein produced OBs at the same level as the control virus and cells had a typical circular morphology. However, cells transfected with recombinant viruses expressing Vpa2Ac1 and Vpa2-like2 lost their rounded shape and became elongated and irregular in appearance. The morphological changes induced by Vpa2Ac1 and Vpa2-like2 were similar to those produced by the microfilament-destabilizing agent cytochalasin D, or those previously described for mammalian cells treated with *C. botulinum* C2 toxin and *C. perfringens* iota toxin [13,14]. These toxins directly interact with actin, interfering with its polymerization and inducing the redistribution of microtubules resulting in the formation of long microtubule-based cell surface extensions [14,33]. The formation of microtubule protrusions is a consequence of actin disruption [12]. We conclude, therefore, that Vpa2Ac1 and Vpa2-like2 are toxins that affect actin polymerization. Moreover, the inclusion of actin filament disrupting agents, such as nicotinamide and nocodazole, during transfection with viruses expressing Vpa2Ac1 and Vpa2-like2 prevented cell polarization and cells recovered their original rounded morphology. In contrast, when the microtubule-stabilizing agent paclitaxel was added, cells showed protrusions. Similarly, Uematsu et al. [13] reported that nocodazole inhibited cell polarization, whereas paclitaxel enhanced polarization. These results are fully consistent with ADP-ribosyltransferase activity in Vpa2Ac1 and Vpa2-like2. Moreover, the fact that those toxins produced actin depolarization in lepidopteran cells suggest that ADP-ribosyltransferase activity is host-independent and that Vpba1/Vpa2 specificity to Coleoptera and Hymenoptera might be due to Vpb1 cell receptors that facilitate Vpa2 binding to brush border membranes of species of those orders. Alternatively, it has been postulated that the specificity of host–pathogen interactions may be due to other virulence factors, unrelated to major classes of protein toxins, such as metalloproteases, chitinases, aminopolyol antibiotics and nucleotide-mimicking moieties [34].

Cells infected with recombinant viruses expressing Vpa2Ac1 and Vpa2-like2 did not produce progeny OBs, as also occurred in baculovirus-infected cells treated with cytochalasin in which actin polymerization was inhibited, resulting in a lack of F-actin and an absence of newly synthesized viral nucleocapsids [35,36]. Cytochalasin interferes with correct nucleocapsid assembly, indicating that microfilaments are involved in this nuclear process [30,35]. However, when actin filament disrupting agents were added, actin polymerization was reversed, and progeny OBs were produced. The actin cytoskeleton is a key component for efficacious baculovirus infection at many stages of the replication cycle [30,36], particularly during virus entry and egress [37,38], and during virus transportation to the nucleus, gene expression and viral progeny production [38,39]. Our results also provide evidence that actin filaments are necessary for normal OB production and toxins/pesticidal proteins that affect actin polymerization indirectly inhibit viral OB production.

Vpa2Ac1 and Vpa2-like2 clearly inhibited the normal replication and production of viral progeny of the recombinant viruses. These two proteins were evidently deleterious for the virus. It is perhaps not surprising, therefore, that the recombinant bacmids rapidly lost these genes from their genome. This was confirmed by REN and PCR analysis (Figure 5). Some genomes lost the recombinant gene after replication in the cell culture, as PCR analysis of BVs showed the presence of two amplification products, one corresponding to the Vpa2Ac1 gene or the Vpa-like2 gene and the other that was the product of the sequences that remained following the loss of these genes in each of the recombinant viruses. In contrast, PCR analysis of the three successive passages (P1-P3) and the OBs obtained after injection of BVs at P0 in larvae revealed that only the product corresponding to Bac-polh-Ø was present, which implies the complete loss of these genes. It has been shown that genes inserted in the genomes of recombinant viruses, to express heterologous proteins at high levels, often prove to be unstable over time and can be rapidly lost [40]. This loss might be particularly rapid when the inserted gene encodes a protein that is deleterious for the virus. Engineered baculoviruses are widely used to express heterologous genes in insect cells, although a large portion of the genomes may appear as defective interfering particles during serial passage [41]. The rapid generation of these particles involves several recombination steps and can prevent the development of stable recombinant baculoviruses [41]. Loss of non-essential genomic regions has been observed to be a frequent phenomenon during virus replication in cell culture and in larvae, presumably because there is no adaptive advantage for the virus to retain such genes [42,43,44]. In the present study, variation in the REN profiles of the Bac-polh-Vpa2Ac1 and Bac-polh-Vpa2like2 recombinant viruses in comparison with the original inocula, suggested that deletions, mutations or recombination events had occurred, resulting in heterogeneous virus populations comprising of mixtures of genotypes. This variability was associated with changes in the biological activity of OBs towards the different lepidopteran species, in which the insecticidal activity of Bac-polh-Vpa2Ac1 and Bac-polh-Vpa2like2 OBs varied across the host species tested. Insects possess both cellular and humoral mechanisms of immunity and both have been implicated in resistance to baculoviruses, which varies markedly across different lepidopteran-alphabaculovirus pathosystems [45]. Moreover, genotypes from the same baculovirus population that differ minimally at the genome level often show different insecticidal characteristics against homologous and heterologous host species [45], i.e., small genetic differences often result in marked variation in the phenotype of these viruses.

It appears that Vpa2-like1 did not display ADP-ribosyltransferase activity. This might be due to differences in the amino acid sequences of Vpa2-like1 and Vpa2Ac1/Vpa2-like2, and that these proteins differ in several domains. For example, the conserved Ser-Thr-Ser motif necessary for NAD binding [46] and the ADP-ribosylating turn-turn motif (ARTT) involved in the polymerization of the actin cytoskeleton [21,23] were absent in Vpa2-like1. Moreover, only one ADP-ribosylation conserved domain at the amino terminal was present in the Vpa2-like1 protein sequence, rather than the two domains present in Vpa2Ac1 and Vpa2-like2. This should result in a non-functional Vpa2-like1 protein (Figure S4), which failed to depolarize actin. However, in lepidopteran larvae, the recombinant virus expressing Vpa2-like1 was less pathogenic than the control virus, in terms of their estimated LC_50_ values. The reason for this was unclear, although Vpa2-like1 might affect viral replication in larvae in some as of yet unknown manner. Further, its divergence from Vip3, Vpb1 and Vpb4 suggests that this protein may be a new class of insecticidal proteins that would require detailed characterization. We are now investigating this and the possible effect of the ATLF super family motif, the anthrax lethal factor, present in the Vpa2-like1 protein.

Finally, the predicted signal peptides were not essential for ADP-ribosyltransferase activity. This can be taken into account when performing toxin optimization prior to plant cell transformation, since Bt toxin truncation has proven to be a useful technique for enhancing toxin activity and improving the levels of expression of the toxin in transgenic crop plants [47].

Vpa2-like2 was identified as a new Vpa2 subfamily, Vpa2C, and has been designated Vpa2Ca1 (accession number AAO86513) by the *Bacillus thuringiensis* delta-endotoxin nomenclature committee [18,19].

## 4. Conclusions

For the first time, we provide several lines of evidence that *B. thuringiensis* Vpa2Ac1 and Vpa2-like2 (designated Vpa2Ca1) proteins exhibited ADP-ribosyltransferase activity leading to cell depolarization in lepidopteran cells, resulting in the same cell morphological changes as observed in other well-documented bacterial insecticidal proteins. Therefore, the specificity of Vpa1/Vpa2 to Coleoptera and Hemiptera is likely due to factors present in Vpa1. This is consistent with Vpa1 being involved in receptor recognition and facilitating the binding of Vpa2 to brush border membrane. This information is relevant when designing new Bt-based insecticides with an extended spectrum of activity.

## 5. Materials and Methods

### 5.1. Vpa2 Protein Analysis

Global alignment, pairwise amino acid sequence comparisons and the best-fit model of amino acid sequence evolution were calculated using Clustal Omega version 1.2.4 (University College, Dublin, Ireland) [48], CLC Genomics Workbench 10.1.1 (QIAGEN, Hilden, Germany) and Protest version 3.4.2 (Department of Biochemistry, Genetics and Immunology, University of Vigo, Vigo, Spain) ([49], respectively. A maximum-likelihood method for phylogenetic analysis was performed using RaxML version 8.2.11 (The Exelixis Lab 2013, Scientific Computing Group, Heidelberg Institute for Theoretical Studies, Schloss-Wolfsbrunnenweg 35, D-69118 Heidelberg, Germany) [50], and the VT model (bootstrap of 10,000 replicates). Well-characterized Vpa, Vpa and Vpb proteins available at https://www.bpprc.org/ (University of Sussex, Cardiff University, University of Florida, CAMTech, NSF) were included in the analysis. Protein domain analysis was performed with NBCI Conserved Domains Search (https://www.ncbi.nlm.nih.gov/Structure/cdd/wrpsb.cgi; Bethesda, MD, USA) and InterProScan tool included in Geneious R11 (https://www.geneious.com, Biomaters, New Zealand) [51]. The sequence data for Vpa2-like1 and Vpa2-like2 proteins described below were deposited in the NCBI GenBank database under accession numbers MT199158 and MT199159, respectively.

### 5.2. Construction of Recombinant Viruses Expressing Vpa2Ac1, Vpa2-like1 and Vpa2-like2 Proteins

Three recombinant viruses were constructed that independently expressed Vpa2 proteins, named Vpa2Ac1, Vpa2-like1 and Vpa2-like2 (accession numbers AAO86513, MT199158 and MT199159, respectively), using a Bac-to-Bac recombination system (Invitrogen^®^, Carlsbard, CA, USA) [52]. Vpa2Ac1 was obtained from the *B. thuringiensis* TF037.2 isolate and was used as a positive reference treatment, as it presented 100% identity with the previously described Vpa2Ac1 [13]. Vpa2-like1 and Vpa2-like2 coding sequences were obtained from *B. thuringiensis* H001.5 and H026.2 genome sequences, respectively. These *B. thuringiensis* strains were isolated from soil samples collected from Tenerife (TF037.2) and El Hierro (H001.5 and H026.2), Canary Islands [29,30]. In order to prevent unpredictable effects of the predicted signal peptide sequences detected with InterProScan [46], the corresponding nucleotide coding sequences were removed from each Vpa2 coding sequence. Therefore, modified Vpa2Ac1, Vpa2-like1 and Vpa2-like2 genes were cloned in the pFastBacTM Dual (pFBD) expression vector under the p10 promoter [47], using the recombinant transfer vector pFBD that included the polyhedrin gene under its own promoter (pFBD-phph-p10x), kindly provided by A. Jakubowska (APIS Applied Insect Science SL, Valencia, Spain). For insertion of the specific modified genes, two pairs of primers were designed to amplify the Vpa2Ac1, Vpa2-like1 and Vpa2-like2 genes. Primers were designed to eliminate the signal peptides (Table 2). The *Xho*I and *Nhe*I restriction sites were introduced near the 5’ termini of the forward and reverse primers, respectively, for further cloning and to direct the transcription of the genes with the p10 promoter. Additionally, a His-tag sequence was included in the C-terminal, for protein purification and detection of the Vpa2Ac1, Vpa2-like1 and Vpa2-like2 proteins.

PCR amplifications were conducted using PrimeStar high fidelity Taq polymerase (Takara, Kusatsu, Shiga, Japan) to obtain fragments of ~1.3 kb, ~1.5 kb and ~1.3 kb for Vpa2Ac1, Vpa2-like1 and Vpa2-like2, respectively (Figure S1A), in agreement with the sizes of these genes of 1287 bp, 1497 bp and 1290 bp, respectively. The DNA fragments were recovered by using the PCR clean-up extraction kit (Macherey-Nagel, Düren, Renania, Germany). The Vpa2Ac1, Vpa2-like1 and Vpa2-like2 genes were cloned into pGEM-T Easy Vector (Promega, Madison, WI, USA) and sequenced by ABI PRISM Big Dye Terminator Cycle Sequencing Ready Reaction Kits with ABI PRISM 3100 Genetic Analyzer (Stab Vida, Caparica, Portugal). After sequence confirmation, clones were digested with *Xho*I and *Nhe*I (New England Biolabs, Ipswich, MA, USA) and the generated restriction fragments were ligated into an *Xho*I-*Nhe*I digested pFBD polyhedrin positive vector. The generated clones were sequenced again in order to confirm the correct insertion of each of the genes under the p10 promoter. Finally, the plasmids were transformed into *Escherichia coli* DH10BTM cells that contained the AcMNPV shuttle vector (bacmid) and the helper plasmid, to produce the recombinant bacmids Bac-polh-Vpa2Ac1, Bac-polh-Vpa2-like1 and Bac-polh-Vpa2-like2, respectively, following transposition of the pFastBac expression construct [52]. To produce a negative control recombinant bacmid (Bac-polh-Ø), the pFBD polyhedrin positive vector, but without any gene of interest under the p10 promoter, was used for transformation.

Colonies were selected on LB (Luria Bertani) agar plaques containing kanamycin (50 µg/mL), gentamycin (7 µg/mL), tetracycline (10 µg/mL), X-gal (100 µg/mL) and IPTG (40 µg/mL). Ten white colonies of each transformant were restreaked on fresh LB agar plates under the same conditions to avoid contamination. Recombinant bacmid DNAs were isolated using the PureLinkTM HiPure Plasmid DNA Miniprep Kit (Invitrogen). The successful transposition to bacmid was analyzed by digestion of DNA with *Pst*I and by PCR amplification and sequence analysis using p10x.F and p10x.R primers that annealed outside the coding region of the p10 promoter in the pFBD vector (Table 2). The PCR product was cloned into pGEM-T Easy Vector and sequenced to confirm the correct transposition of the Vpa2Ac1, Vpa2-like1 and Vpa2-like2 genes.

Correct insertion of these genes was confirmed by restriction endonuclease (REN) analysis (Figure S1B) and sequencing analysis after PCR amplification of adjacent regions using the p10x.F and p10.R primers (Figure S1C). Sequence analysis confirmed that Vpa2Ac1, Vpa2-like1 and Vpa2-like2 genes were inserted after the p10 promoter in the correct translational phase for expression. Following this, the corresponding DNAs were purified and transfected into insect cells for the production of viral occlusion bodies (OBs).

### 5.3. Transfection of Sf9 Cells with Recombinant Viruses

Recombinant baculoviruses were produced by transfecting 1 μg of Bac-polh-Ø, Bac-polh-Vpa2Ac1, Bac-polh-Vpa2like1 and Bac-polh-Vpa2like2 genomic DNAs into Sf9 cells using Lipofectin^®^ reagent (Invitrogen) [53]. The production of OBs within the cells was checked daily and at 5 days post-infection, the supernatant containing budded virus (BV) and the pellet containing the cells and viral occlusion bodies (OBs) were recovered. DNA extraction was performed from infected cells [53] to confirm the restriction endonuclease (REN) profiles of the recombinant bacmids and to check for cross contamination.

Finally, recombinant bacmid OBs were produced by injecting 8 μL of BV suspension of each virus at 1:1000 dilution into *Spodoptera exigua* fifth instar larvae from a healthy laboratory colony. The fidelity of OBs produced in insects was also confirmed by REN analysis after DNBA extraction from OBs [54] and by sequencing of the PCR products obtained following amplification using p10x.F and p10x.R primers (Table 2).

### 5.4. Detecting Vpa2Ac1, Vpa2-like1 and Vpa2-like2 Transcripts and Proteins in Transfected Cells

Transcription of Vpa2Ac1, Vpa2-like1 and Vpa2-like2 genes was confirmed by RT-PCR amplification of RNA extracted 12, 24, 48 and 72 h after transfection with recombinant DNAs in 6-well dishes. Transfected Sf9 cells were removed by pipetting, pelleted by centrifugation and resuspended in 500 µL PBS. Total RNA was isolated using the Trizol^®^ Reagent (Invitrogen) following the manufacturer’s instructions. RNA quantification was performed using a spectrophotometer (NanoDrop, ThermoFisher, Waltham, MA, USA). The synthesis of cDNA was performed by reverse transcription on 2 μg of RNA using a reverse transcriptase (Promega) with reverse primer oligo(dT) anchor primer. An aliquot of the reaction was then subjected to PCR amplification using primers located upstream from the stop codons (Table 2).

Additionally, a Western blot was performed to detect Vpa2Ac1, Vpa2-like1 and Vpa2-like2 proteins in infected cells using specific antibodies for His-tagged proteins (Sigma-Aldrich, Seeize, Germany), as the C-terminal region of the proteins included a His-tag tail. Cells transfected with the recombinant viruses were incubated for 5 days, removed and suspended in TC100 medium, and used for the detection of the proteins by Western blotting. A 1 mL volume of each cell sample was centrifuged at 5000 rpm during 5 min and the pellet resuspended in 50 µL of 1×SDS loading buffer. Samples were boiled for 10 min and centrifuged again at 5000 rpm during 5 min. A 10 µL volume of the top viscous extract was used to load the SDS-PAGE gel [55]. Samples were separated by 7.5% SDS-PAGE at 150 V, followed by electroblotting to a nitrocellulose membrane for 2 h at 100 V (BioRad, Hercules, CA, USA). Membranes were probed with anti-His tag antibody (1:1000) and incubated 1 h at RT. Bound antibodies were detected using the chemiluminescence in a ChemiDoc imaging system (Bio-Rad, Hercules, CA, USA). No cross-reactivity was observed, but different exposure times were used for the detection of the specific isoforms due to differences in expression levels.

### 5.5. Effects of Actin-Affecting Agents on Cell Morphology

The microfilament destabilizing agent, Cytochalasin D (Thermo Fisher Scientific) that inhibits actin polymerization and two filament disrupting agents, Nicotinamide (N0636, Sigma-Aldrich) and Nocodazole (M1404, Sigma-Aldrich), and the filament-stabilizing agent, Paclitaxel (T7402, Sigma-Aldrich) were used in this assay. Cytochalasin D was added to non-infected cells at a final concentration of 0.5 µM with TC-100 medium and serum, as the control to examine the cell morphology produced by an actin destabilizing agent.

For disrupting and stabilizing agents, 5 × 10^5^ cells were transfected with recombinant DNAs, as described above, and after transfections, filament disrupting and stabilizing agents were added at concentrations of 100 nM, 500 nM and 1 µM to TC-100 medium containing serum and antibiotics. Cells were incubated at 25 °C for 10 days and checked daily to examine cell morphology and the presence of viral OBs in cell nuclei as an indicator of productive virus infection. After that, cells were harvested and centrifuged at 5000 rpm for 5 min, to separate cell pellets and BVs (P0) (supernatant).

Bac-polh-Vpa2Ac1, Bac-polh-Vpa2like1, Bac-polh-Vpa2like2 and Bac-polh-Ø BVs at P0 obtained after transfection with nicotinamide and nocodazole were subjected to three passages in Sf9 cell culture (P1-P3). To achieve this, at each passage 500 µL of BV suspension, obtained as described above, were used for successive infections of 1 × 10^6^ cells in a 25 cm^2^ flask. At each passage, 8 μL volume of BV suspension at 1:1000 dilution was used to inject 30 *S. exigua* fifth instar larvae to evaluate the stability of the recombinant viruses. For this, OBs recovered from larvae at each passage and for each virus were purified and DNA was extracted and analyzed by REN [54] and PCR using p10x.F and p10x.R primers (Table 2).

### 5.6. Determining the Insecticidal Characteristics of Recombinant Viruses

To determine the insecticidal characteristics of each virus, OBs were produced in large quantities in insects, as those obtained in cell culture were insufficient for insect bioassays. For this, *S. exigua* larvae were injected with the BVs at P0, obtained after transfection of Sf9 cells with recombinant bacmids Bac-polh-ø, Bac-polh-Vpa2Ac1, Bac-polh-Vpa2like1 and Bac-polh-Vpa2like2, and BVs obtained from cells treated with nicotinamide in the case of Bac-polh-Vpa2Ac1 and Bac-polh-Vpa2like2. The pathogenicity of OBs was compared in terms of 50% lethal concentration (LC_50_) with the OBs of the control virus that did not express any recombinant gene of interest, Bac-polh-Ø.

Bioassays were performed following the droplet feeding method [56], on second instars of *Mamestra brassicae*, *S. exigua, Spodoptera littoralis* and *Spodoptera frugiperda*. Groups of larvae of each species were starved for 8–12 h at 25 °C and were then allowed to drink from an aqueous suspension containing 10% (*w*/*v*) sucrose, 0.001% (*w*/*v*) Fluorella blue and one of five concentrations of OBs. For *M. brassicae* and *S. littoralis* larvae, the concentrations applied were 1 × 10^8^, 2 × 10^7^, 4 × 10^6^, 8 × 10^5^ and 1.6 × 10^5^ OBs/mL, whereas for *S. exigua* and *S. frugiperda* inoculum concentrations were 2 × 10^7^, 5 × 10^6^, 1 × 10^6^, 2 × 10^5^ and 4 × 10^4^ OBs/mL. These concentrations were previously determined to kill between 95% and 5% of the experimental insects for each of the species tested. Larvae that ingested the suspension within 10 min were transferred individually to the wells of a 28-well dish with a piece of semi-synthetic formaldehyde-free diet [56]. Bioassays were performed three times using groups of 28 larvae per virus concentration and 28 mock-infected control larvae. Larvae were reared at 26 ± 1 °C and mortality was recorded daily until insects had either died or pupated. Virus-induced mortality results were subjected to probit analysis using the Polo-Plus program [57].

## Figures and Tables

**Table 1:** LC_50_ values (lethal concentration that kills 50% of the population assayed), relative potencies and fiducial limits of the relative potencies of Bac-polh-ø (control virus), Bac-polh-Vpa2Ac1, Bac-polh-Vpa2like1 and Bac-polh-Vpa2like2 OBs in second instar larvae of four lepidopteran species.

Insect Species	Virus	LC_50_ (OBs/mL)	Relative Potency	Fiducial Limits (95%)	
				Lower	Upper
*M. brassicae*	Bac-polh-ø	6.79 × 10^6^	1	-	-
	Bac-polh-Vpa2Ac1	1.23 × 10^7^	0.55	0.35	0.87
	Bac-polh-Vpa2like1	2.36 × 10^7^	0.29	0.17	0.50
	Bac-polh-Vpa2like2	6.51 × 10^6^	1.04	0.66	1.64
*S. exigua*	Bac-polh-ø	2.97 × 10^5^	1	-	-
	Bac-polh-Vpa2Ac1	6.18 × 10^5^	0.48	0.30	0.76
	Bac-polh-Vpa2like1	1.00 × 10^5^	0.30	0.19	0.47
	Bac-polh-Vpa2like2	6.14 × 10^5^	0.48	0.30	0.77
*S. littoralis*	Bac-polh-ø	1.24 × 10^7^	1	-	-
	Bac-polh- Vpa2Ac1	1.47 × 10^7^	0.84	0.50	1.40
	Bac-polh-Vpa2like1	3.17 × 10^7^	0.39	0.23	0.68
	Bac-polh-Vpa2like2	1.107 × 10^7^	1.13	0.70	1.82
*S. frugiperda*	Bac-polh-ø	8.43 × 10^5^	1	-	-
	Bac-polh- Vpa2Ac1	1.03 × 10^6^	0.82	0.53	1.24
	Bac-polh-Vpa2like1	4.83 × 10^6^	0.18	0.12	0.26
	Bac-polh-Vpa2like2	1.53 × 10^6^	0.55	0.35	0.86

Probit regressions were fitted in POLO Plus. A test for non-parallelism was not significant for *M. brassicae* (χ^2^ = 5.02; d.f = 3; *p* = 0.17), *S. exigua* (χ^2^ = 0.36; d.f = 3; *p* = 0.95), *S. littoralis* (χ^2^ = 2.31; d.f = 3; *p* = 0.51), or *S. frugiperda* (χ^2^ = 6.66; d.f = 3; *p* = 0.083). Relative potency was calculated as the ratio of LC_50_ values with reference to the Bac-polh-ø control virus in all cases.

**Table 2:** Primers used in this study.

Primer	Sequence (Position in the Genome)	Amplification Purpose
BB.Vpa2Ac1-F	5′-CCGCTCGAGATGCATCATCATCATCATCACAATTCTCAAAATAAATATAC-3′	*Vpa2Ac1* amplification. TF037.2 isolate was used as template. Underlined the *Xho*I site and Hig-tag sequence.
BB.Vpa2Ac1-R	5′-CTAGCTAGCTTAATTTGTTAATAATGTTGCATCCACTACATATCGCTTAAC-3′	*Vpa2Ac1* amplification. TF037.2 isolate was used as template. Underlined the *Nhe*I site.
BB.Vpa2like1-F	5′-CCGCTCGAGATGCATCATCATCATCATCACGAGAATTGGGATCCAATAAG-3′	*Vpa2-like1* amplification. H001.5 isolate was used as template. Underlined the *Xho*I site and Hig-tag sequence
BB.Vpa2like1-R	5′-TAGCTAGCTTATTTTTTCGCTAGAATGGAAGGTGAACTAGCGTTTATTTC-3′	*Vpa2-like1* amplification. H001.5 isolate was used as template. Underlined the *Nhe*I site.
BB.Vpa2like2-F	5′-CCGCTCGAGATGCATCATCATCATCATCACTATTCTGGTAAGAAACTAAATC-3′	*Vpa2-like2* amplification. H26.2 isolate was used as template. Underlined the *Xho*I site and Hig-tag sequence.
BB.Vpa2like2-R	5′-CTAGCTAGCCTATCTTGTTAGCAAAGTTGCATCTACTATATATCTCTTGACG-3′	*Vpa2-like1* amplification. H26.2 isolate was used as template. Underlined the *Nhe*I site.
p10x.F	5’-GTAGATCTTGTTGTCGTACA-3’	Forward primer that annealed outside the coding region of the p10 promoter (within the *polyhedrin* gene) in the pFBD-phph-p10x.
p10x.R	5’-TTATTGCCGTCATAGCGCGG-3’	Reverse primer that annealed outside the coding region of the p10 promoter in the pFBD-phph-p10x.
Vpa2Ac1-R	5’-CAATCAAAGAAGACAAAGGA-3’	Reverse primer located 260 nt upstream of the *Vpa2Ac1* stop codon. For *Vpa2Ac1* transcript detection.
Vpa2-like1-R	5’-ATTCAGGCGTTTCGAATATG-3’	Reverse primer located 242 nt upstream of the *Vpa2-like1* stop codon. For *Vpa2-like1* transcript detection.
Vpa2-like2-R	5’- TTAGATACAATTAAGGATGA -3’	Reverse primer located 267 nt upstream of the *Vpa2-like2* stop codon. For *Vpa2-like1* transcript detection.

## Supplementary Materials

The following are available online at https://www.mdpi.com/2072-6651/12/9/543/s1, Figure S1: (A) PCR amplification of *vpa2Ac1*, *vpa2-like1* and *vpa2-like2* genes from *B. thuringiensis*. The molecular size marker (M) was smart ladder (Stratagene). (B) Restriction endonuclease profiles with *Pst*I of three replicates of each recombinant bacmid; Bac-polh-Vpa2Ac1, Bac-polh-Vpa2like1, Bac-polh-Vpa2like2 and Bac-polh-ø. White stars indicate diagnostic restriction fragments. The molecular size marker (M) was smart ladder (Stratagene). (C) Sequence analysis of the PCR products generated with p10x.F and p10x.R primers for each recombinant bacmid that included *vpa2Ac1*, *vpa2-like1* and *vpa2-like2* genes., Figure S2: Restriction endonuclease profiles with *Pst*I of (a) cell pellets of four transfection replicates in Sf9 cells with recombinant bacmids Bac-polh-ø, Bac-polh-Vpa2Ac1, Bac-polh-Vpa2like1 and Bac-polh-Vpa2like2, and (b) OBs obtained after injection of budded virus in transfection supernatant into *S. exigua* fourth instar larvae. OBs were only obtained for Bac-polh-ø and Bac-polh-Vpa2like1 transfection supernatant. In both images white stars indicate diagnostic RFLP fragments. The molecular size marker (M) was smart ladder (Stratagene), Figure S3: (a) RT-PCR analysis of *vpa2Ac1*, *vpa2-like1* and *vpa2-like2* genes of recombinant Bac-polh-Vpa2Ac1 (Ac1), Bac-polh-Vpa2like1 (like1) and Bac-polh-Vpa2like2 (like2) viruses performed on total RNA extracted from Sf9 cells at 12 and 24 hpi post-transfection. The molecular size marker, M, used was smart ladder (Stratagene). Ø indicates the result of RT-PCR on total RNA extracted from cells infected with Bac-polh-ø using oligo(dT) and Vpa2Ac1-R; no amplification was obtained when Vpa2-like1-R and Vpa2-like2-R primers (Table 2) were used (data not shown); (b) Western-blot analysis to detect Vpa2Ac1, Vpa2-like1 and Vpa2-like2 proteins in infected cells using anti His-tag specific antibodies. Cells were transfected with Bac-polh-ø (Ø), Bac-polh-Vpa2Ac1 (Ac1), Bac-polh-Vpa2like1 (like1) and Bac-polh-Vpa2like2 (like2) viruses, and 5 days after transfection a 10 µL sample of transfected cells was subjected to SDS-PAGE, followed by electroblotting to a nitrocellulose membrane. Membranes were probed with anti His-tag antibody and detected using chemiluminescence. M indicates molecular size marker, the Precision Plus Protein All Blue from Bio-Rad. An empty lane (Emp) was left to avoid possible contamination with the signal produced by the molecular marker, Figure S4: Multiple sequence alignment of full-length Vpa2Ac protein (Acc. No. AY245547) and modified Vpa2Ac1, Vpa2-like1 and Vpa2-like2 proteins performed with MAFFT [58]. InterProScan conserved domains are indicated with light blue and green rectangles.
