# Supplementary material for: Baculovirus Expression and Functional Analysis of Vpa2 Proteins from Bacillus thuringiensis
Source: Toxins (Basel). 2020 Aug 22;12(9):543. doi: 10.3390/toxins12090543 (PMC7551607; doi:10.3390/toxins12090543)
Supplement: Supplementary file 1 [file toxins-12-00543-s001.pdf]

Supplementary Materials: Baculovirus Expression and Functional Analysis of Vpa2 Proteins from *Bacillus thuringiensis*

Oihane Simón, Leopoldo Palma, Ana Beatriz Fernández , Trevor Williams and Primitivo Caballero

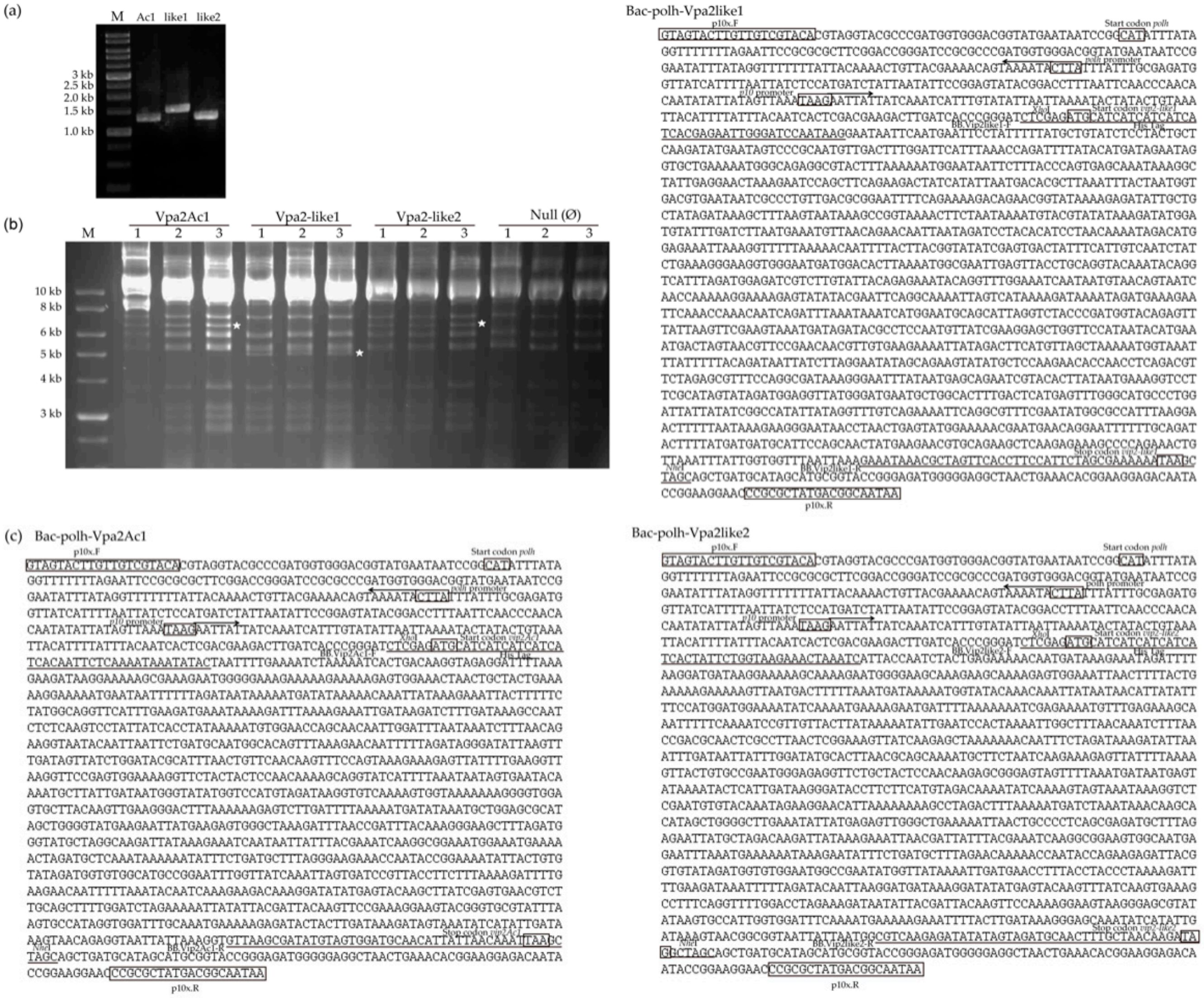

Figure S1. (**A**) PCR amplification of *vpa2Ac1*, *vpa2-like1* and *vpa2-like2* genes from *B. thuringiensis*. The molecular size marker (M) was smart ladder (Stratagene) (**B**) Restriction endonuclease profiles with *Pst*I of three replicates of each recombinant bacmid; Bac-polh-Vpa2Ac1, Bac-polh-Vpa2like1, Bac-polh-Vpa2like2 and Bac-polh-ø. White stars indicate diagnostic restriction fragments. The molecular size marker (M) was smart ladder (Stratagene). (**C**) Sequence analysis of the PCR products generated with p10x.F and p10x.R primers for each recombinant bacmid that included *vpa2Ac1*, *vpa2-like1* and *vpa2-like2* genes.

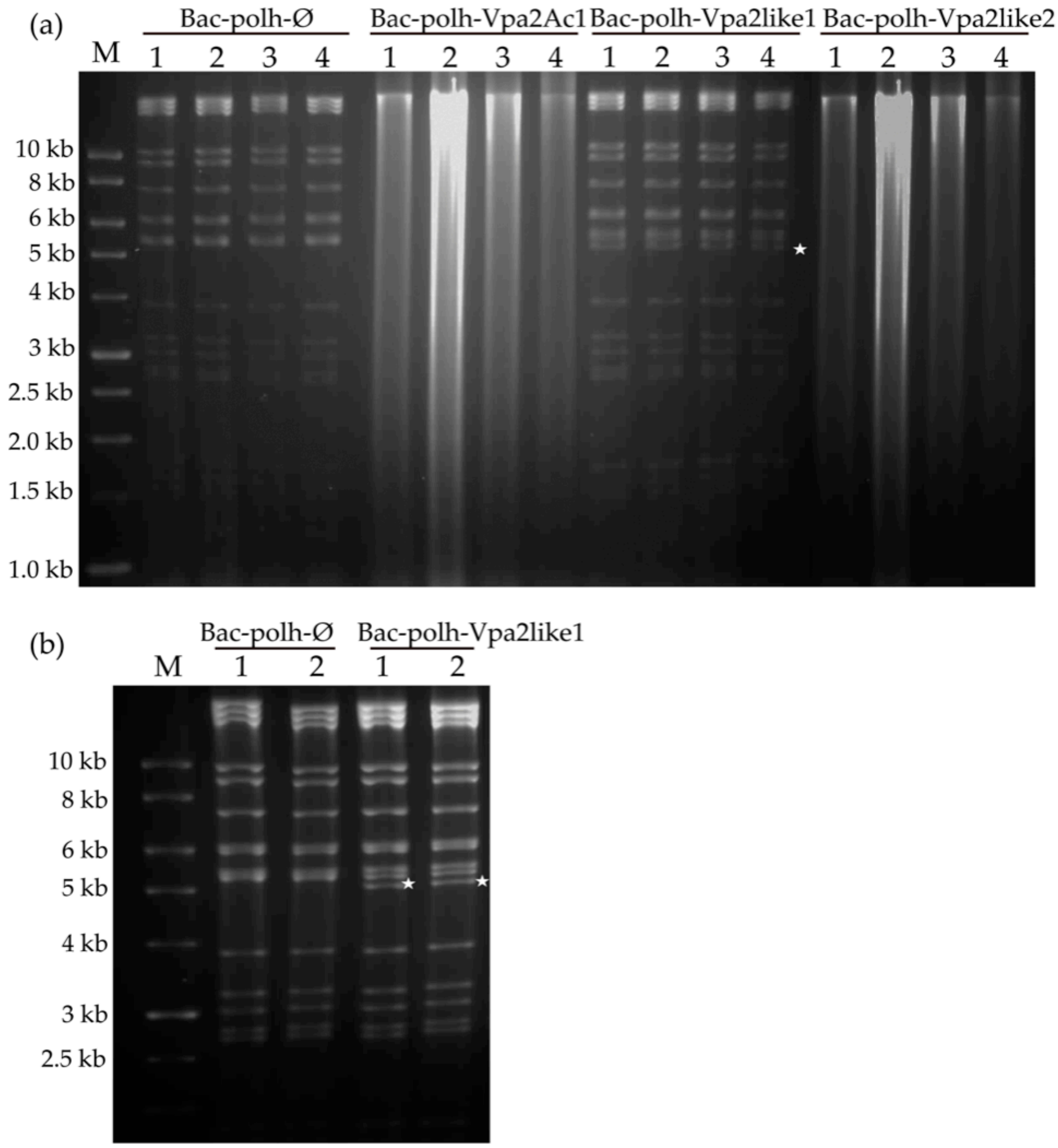

Figure S2. Restriction endonuclease profiles with *Pst*I of (**a**) cell pellets of four transfection replicates in Sf9 cells with recombinant bacmids Bac-polh-ø, Bac-polh-Vpa2Ac1, Bac-polh-Vpa2like1, and Bac-polh-Vpa2like2, and (**b**) OBs obtained after injection of budded virus in transfection supernatant into *S. exigua* fourth instar larvae. OBs were only obtained for Bac-polh-ø and Bac-polh-Vpa2like1 transfection supernatant. In both images white stars indicate diagnostic RFLP fragments. The molecular size marker (M) was smart ladder (Stratagene).

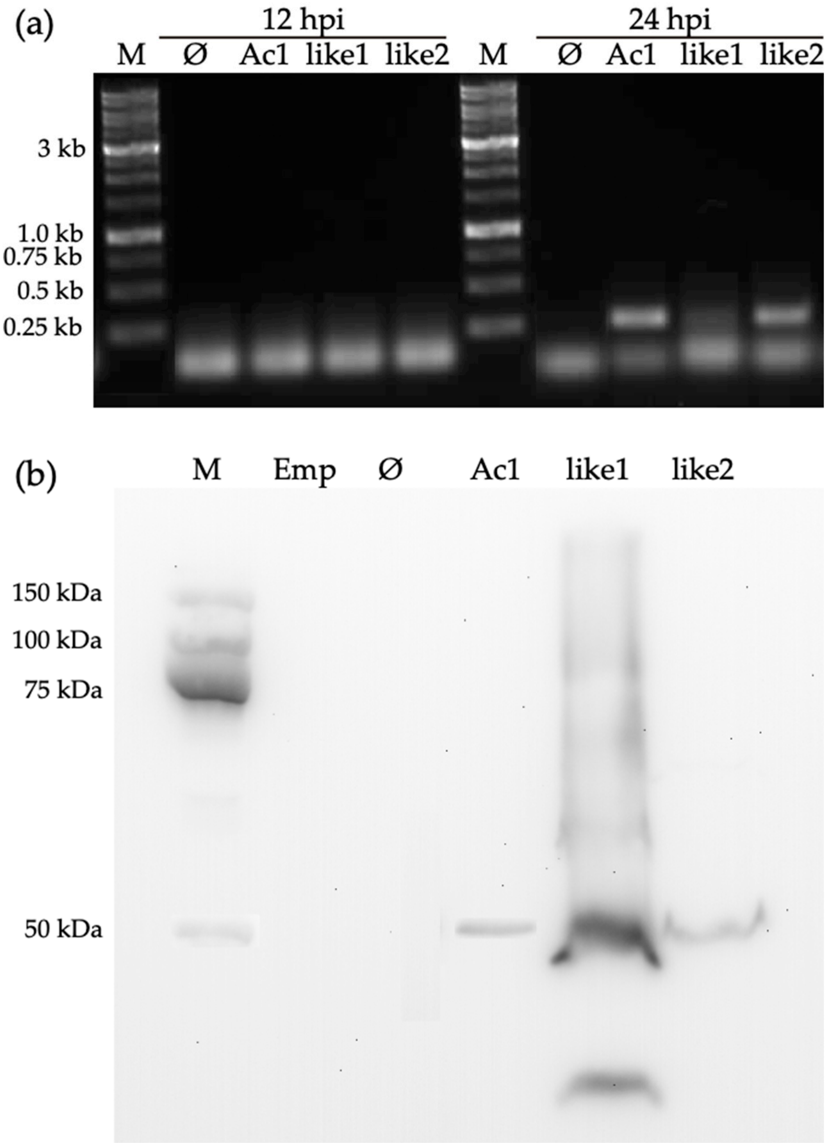

Figure S3. (**a**) RT-PCR analysis of *vpa2Ac1*, *vpa2-like1* and *vpa2-like2* genes of recombinant Bac-polh-Vpa2Ac1 (Ac1), Bac-polh-Vpa2like1 (like1) and Bac-polh-Vpa2like2 (like2) viruses performed on total RNA extracted from Sf9 cells at 12 and 24 hpi post-transfection. The molecular size marker, M, used was smart ladder (Stratagene). Ø indicates the result of RT-PCR on total RNA extracted from cells infected with Bac-polh-ø using oligo(dT) and Vpa2Ac1-R; no amplification was obtained when Vpa2-like1-R and Vpa2-like2-R primers (Table 2) were used (data not shown); (**b**) Western-blot analysis to detect Vpa2Ac1, Vpa2-like1 and Vpa2-like2 proteins in infected cells using anti His-tag specific antibodies. Cells were transfected with Bac-polh-ø (Ø), Bac-polh-Vpa2Ac1 (Ac1), Bac-polh-Vpa2like1 (like1) and Bac-polh-Vpa2like2 (like2) viruses, and 5 days after transfection a 10 µl sample of transfected cells was subjected to SDS-PAGE, followed by electroblotting to a nitrocellulose membrane. Membranes were probed with anti His-tag antibody and detected using chemiluminescence. M indicates molecular size marker, the Precision Plus Protein All Blue from Bio-Rad. An empty lane (Emp) was left to avoid possible contamination with the signal produced by the molecular marker.

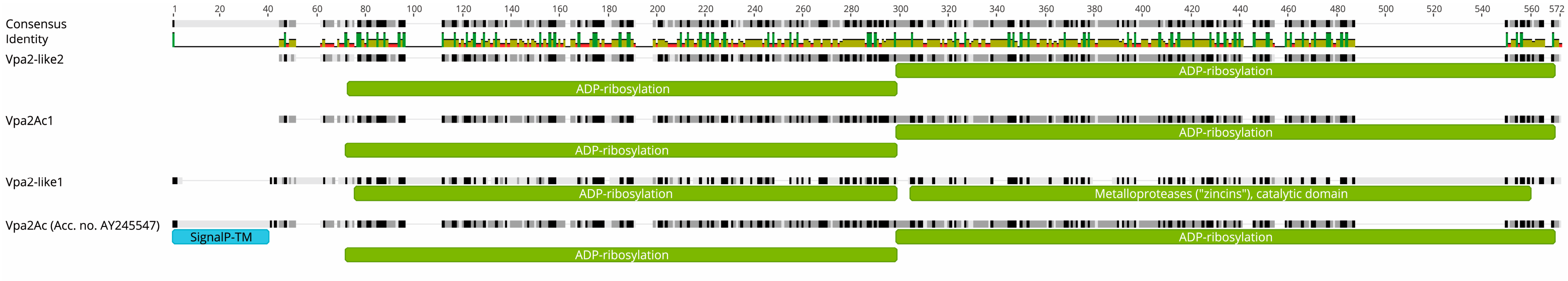

Figure S4. Multiple sequence alignment of full-length Vpa2Ac protein (Acc. No. AY245547) and modified Vpa2Ac1, Vpa2-like1 and Vpa2-like2 proteins performed with MAFFT [58]. InterProScan conserved domains are indicated with light blue and green rectangles.
